# Genetic analysis of harvest samples reveals population structure in a highly mobile generalist carnivore

**DOI:** 10.1002/ece3.11411

**Published:** 2024-05-23

**Authors:** Stuart C. Fetherston, Robert C. Lonsinger, Lora B. Perkins, Chadwick P. Lehman, Jennifer R. Adams, Lisette P. Waits

**Affiliations:** ^1^ Natural Resource Management South Dakota State University Brookings South Dakota USA; ^2^ U.S. Geological Survey, Oklahoma Cooperative Fish and Wildlife Research Unit Oklahoma State University Stillwater Oklahoma USA; ^3^ South Dakota Department of Game, Fish and Parks, Custer State Park Custer South Dakota USA; ^4^ Fish and Wildlife Sciences University of Idaho Moscow Idaho USA; ^5^ Present address: U.S. Fish and Wildlife Service, Texas Fish and Wildlife Conservation Office San Marcos Texas USA

**Keywords:** barriers, bobcat, carnivore, genetic structure, harvest, *Lynx rufus*, management units, population genetics

## Abstract

Delineating wildlife population boundaries is important for effective population monitoring and management. The bobcat (*Lynx rufus*) is a highly mobile generalist carnivore that is ecologically and economically important. We sampled 1225 bobcats harvested in South Dakota, USA (2014–2019), of which 878 were retained to assess genetic diversity and infer population genetic structure using 17 microsatellite loci. We assigned individuals to genetic clusters (*K*) using spatial and nonspatial Bayesian clustering algorithms and quantified differentiation (*F*_ST_ and $G_{\mathrm{ST}}^{\prime \prime }$) among clusters. We found support for population genetic structure at *K* = 2 and *K* = 4, with pairwise *F*_ST_ and $G_{\mathrm{ST}}^{\prime \prime }$ values indicating weak to moderate differentiation, respectively, among clusters. For *K* = 2, eastern and western clusters aligned closely with historical bobcat management units and were consistent with a longitudinal suture zone for bobcats previously identified in the Great Plains. We did not observe patterns of population genetic structure aligning with major rivers or highways. Genetic divergence observed at *K* = 4 aligned roughly with ecoregion breaks and may be associated with environmental gradients, but additional sampling with more precise locational data may be necessary to validate these patterns. Our findings reveal that cryptic population structure may occur in highly mobile and broadly distributed generalist carnivores, highlighting the importance of considering population structure when establishing population monitoring programs or harvest regulations. Our study further demonstrates that for elusive furbearers, harvest can provide an efficient, broad‐scale sampling approach for genetic population assessments.

## Graphical abstract

Delineating wildlife population boundaries is important for effective population monitoring and management. We sampled harvested bobcats in South Dakota, USA, to infer population genetic structure and inform management. Our findings indicated that despite having a broad distribution, high movement capacity, and generalist tendencies, bobcats in South Dakota were genetically differentiated into eastern and western populations, with preliminary evidence of further differentiation within the western population.

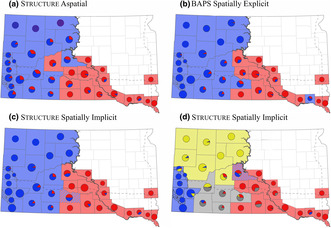

## 1 INTRODUCTION

Management units for regulating wildlife harvest are often defined based on geographical features (e.g., watersheds), political boundaries, or other features that are easily identified by hunters or trappers (Connelly et al., 2012). However, this approach may not consider population units and may lead to discrepancies between the scale of populations and the scale of management (Conner & Miller, 2004). Large management units encompassing multiple discrete populations could lead to overharvesting of some populations, even if the overall harvest is low, if harvest within a unit is distributed unevenly (e.g., harvest is concentrated on a specific population; Taylor & Dizon, 1999). In contrast, small management units may not sufficiently encompass the area required to support a population (Rosenberry & Diefenbach, 2019) and may lead to increased monitoring or management costs (Allendorf et al., 2013). Therefore, it has been recommended that management units be delineated through the identification of natural (or independent) populations for which population dynamics depend more on local reproduction and mortality than metapopulation dynamics (Palsbøll et al., 2007).

The delineation of wildlife population boundaries is difficult but necessary to inform effective population monitoring (e.g., abundance, demographic rates, and population growth; Nichols et al., 1992; Otis et al., 1978); disease management (Côté et al., 2012; DeYoung et al., 2009); habitat management (Klaver et al., 2008); and harvest management (Bethke et al., 1996). Mark‐recapture and telemetry have been used to identify wildlife population boundaries (Amstrup et al., 2004; McLoughlin et al., 2002; Royle et al., 2013; Viengkone et al., 2018), but they require intensive data collection that may be prohibitive over broad spatial scales (Rushing et al., 2016; Zipkin et al., 2014). Count‐based approaches for delineating populations from unmarked animals may be more feasible over broad scales (Rushing et al., 2016) but may not be effective for elusive species. Approaches such as modeling habitat connectivity (Dickson et al., 2013) may not adequately characterize gene flow, which then may be misleading if used to delineate population boundaries (Koenig et al., 1996; Mateo‐Sánchez et al., 2015). Landscape features and heterogeneity that influence gene flow by impeding dispersal can produce population genetic structure; for example, dispersal may be limited by geographic distances (i.e., isolation by distance; Wright, 1942), absolute or permeable barriers (i.e., isolation by barrier or resistance, respectively; McRae, 2006), or differences among environments (i.e., isolation by environment; Wang & Bradburd, 2014). Consequently, population genetic structure can provide an alternative approach to identify independent populations and inform management units (Moritz, 1994).

Genetic sampling of wildlife introduces its own challenges. Noninvasive genetic sampling can facilitate efficient, broad‐scale sampling of elusive carnivores (Waits & Paetkau, 2005). For example, grizzly bear (*Ursus arctos*; Kendall et al., 2008) and wolverine (*Gulo gulo*; Brøseth et al., 2010) populations have been assessed at broad scales using hair and fecal DNA, respectively. For widely distributed species with high dispersal capacities, such as many carnivores, gene flow is often predicted to be sufficiently high to limit genetic divergence at local scales (i.e., panmixia, or no population structure) or result in patterns of isolation by distance at broad scales (Sunquist & Sunquist, 2001). Similarly, for highly mobile generalist species, population boundaries may be cryptic (Rowe & Beebee, 2007). Despite these predictions and challenges, many carnivore populations have exhibited fine‐scale patterns of population genetic structure associated with anthropogenic (e.g., roads; Riley et al., 2006) or natural (e.g., rivers; Hartmann et al., 2013) barriers, reduced landscape permeability due to unfavorable land‐cover types (McRae et al., 2005), or macro‐ (Sacks et al., 2004) or micro‐ (Lonsinger et al., 2015) environmental differences in the absence of substantial barriers or distances between populations.

**FIGURE 1:**
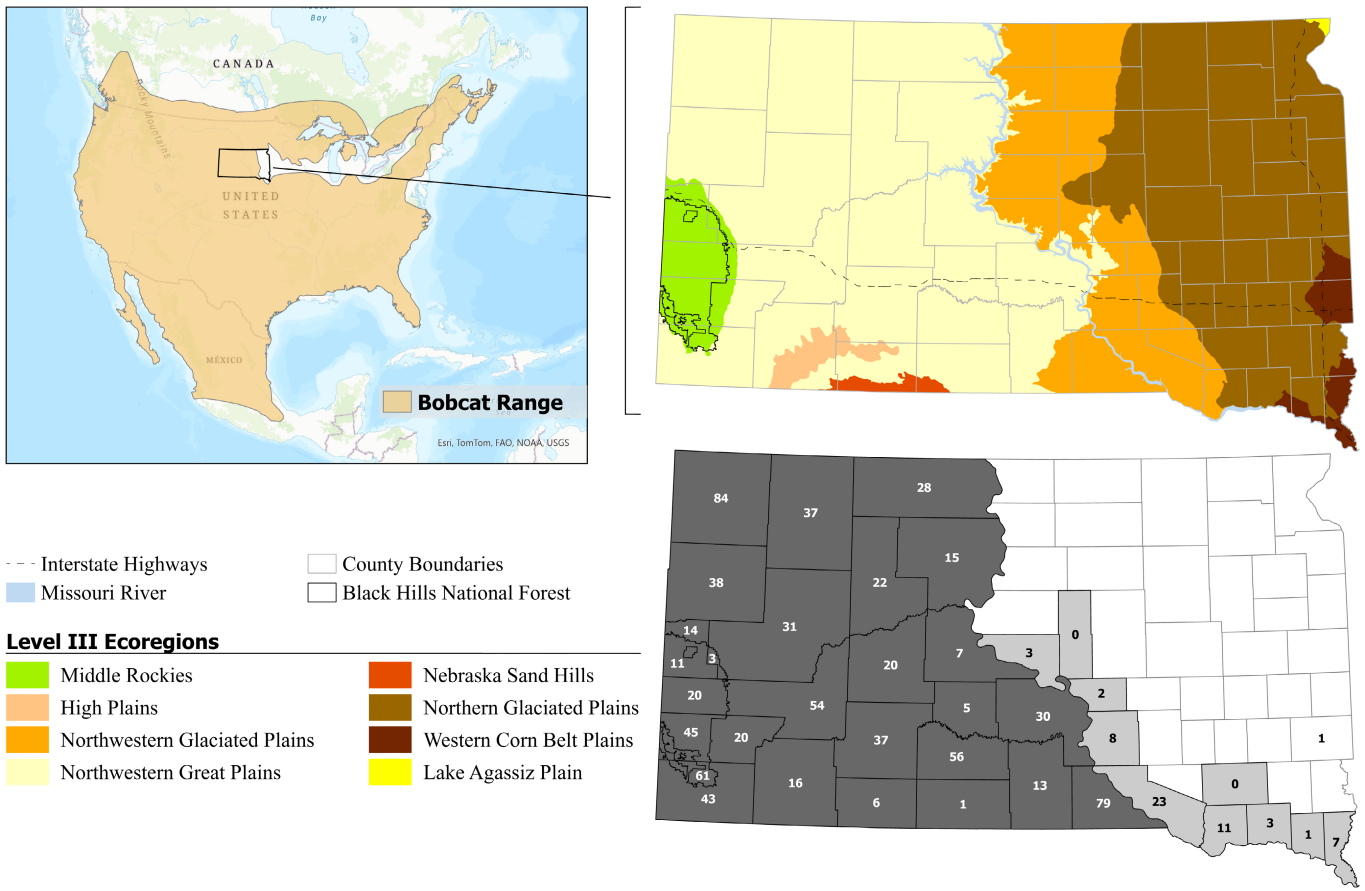
(Top left) Range of bobcats (*Lynx rufus*; International Union for Conservation of Nature, 2016) with respect to the location of South Dakota (USA), (Top right) level III ecoregions (U.S. Environmental Protection Agency, 2013) and linear landscape features, and (Bottom right) counties (or subcounties) in which bobcat harvest was open (west of the Missouri River = dark gray; east of the Missouri River = light gray) or closed (white) from December 2014 to February 2019, and sample size contributed to spatial analyses of population genetic structure; one sample was provided from a county closed to harvest and 23 additional samples lacked spatial data and were retained for aspatial analyses. The basemap for North America was obtained from the Esri World Topographic Map, whereas South Dakota state and county boundaries were acquired from the South Dakota Global Information Systems Data (https://opendata2017‐09‐18t192802468z‐sdbit.opendata.arcgis.com/).

Bobcats (*Lynx rufus*) are generalist carnivores that exploit a wide range of land‐cover types (Anderson & Lovallo, 2003), have high dispersal capabilities (>150 km; Knick & Bailey, 1986), and are broadly distributed across North America (Roberts & Crimmins, 2010; Woolf & Hubert, 1998). In the United States, there is a substantial gap in the bobcat distribution in the Midwest region from which bobcats were extirpated following land‐use changes in the mid‐1800s to early 1900s (Figure 1; Woolf & Hubert, 1998). Bobcat populations are presumably stable (or increasing) and not severely fragmented, with natural recolonization occurring in areas from which they were extirpated (Anderson et al., 2015; Bauder et al., 2023; Hughes et al., 2019; Roberts & Crimmins, 2010). Consequently, bobcats are classified as a species of “least concern” by the International Union for Conservation of Nature (IUCN; Kelly et al., 2016). Bobcat harvest is closely regulated in the United States and Canada, and the Convention of the International Trade in Endangered Species of Wild Fauna and Flora (CITES) regulates international trade of bobcat pelts under appendix II, which is the designation for all *Felidae* that are not regulated under appendix I for conservation concerns (CITES, 2024). Management units for regulating bobcat harvest are often large, presumably due to the large movement capacity of bobcats and limited resources for management. Although bobcats are panmictic in some regions (Croteau et al., 2010; Reid, 2006), evidence suggests bobcat movement and gene flow may be restricted by mountains (Reding et al., 2013), rivers (Nielsen & Woolf, 2003), highways (Litvaitis et al., 2015; Riley et al., 2006), or anthropogenic landcover (Reed et al., 2017; Smith et al., 2020).

Here, we quantified the population genetic structure of bobcats in South Dakota to delineate populations and inform management of a highly mobile and elusive generalist carnivore. In South Dakota, bobcat management units were initially defined in 1977 by separating the state along the Missouri River into western and eastern management units, with harvest permitted in the west but closed in the east where bobcats were rare or absent (within the Midwest distributional gap; Tycz, 2016; Figure 1). Following research indicating that bobcats were common in limited portions of eastern South Dakota (Mosby, 2011), harvest was established in select counties east of the Missouri River in 2012 with a shorter harvest season and a limited (i.e., bag limit = 1) harvest (Figure 1). However, it is unclear whether these management units accurately reflect the population structure of bobcats in South Dakota. Our objective was to test the null hypothesis that bobcats (in portions of South Dakota with bobcat harvest) represented a single, panmictic population. Alternatively, we hypothesized that patterns of population genetic structure (i.e., population differentiation) would be associated with putative linear barriers to dispersal (i.e., major rivers and highways), habitat‐specific breaks that may influence dispersal tendencies, or a combination of these factors. We predicted that if rivers influenced dispersal, bobcat populations on the western and eastern sides of the Missouri River (the largest river in South Dakota) would be differentiated from one another. Similarly, we predicted that if major highways influenced bobcat dispersal, population boundaries would closely align with a heavily trafficked highway (i.e., Interstate 90) that traversed South Dakota. Finally, we predicted that if habitat‐specific breaks influenced bobcat dispersal, bobcats in the Black Hills would be a discrete population due to the distinctiveness of the habitat in the Black Hills relative to other portions of the state.

## 2 METHODS

### 2.1 Study area

The study area included South Dakota counties with bobcat harvests between December 2014 and February 2019 and one county where South Dakota Game, Fish and Parks (SDGFP) officials collected a nonharvest bobcat sample (Figure 1). During sample collection, the bobcat harvest in South Dakota was divided into the following three management units: (1) harvest was permitted with no bag limit from late December through mid‐February in the 22 counties west of the Missouri River; (2) a limited harvest (i.e., bag limit = 1) was permitted in 10 counties east of the Missouri River over a shorter harvest season from late December through mid‐January; and (3) harvest was not permitted in the remaining 34 counties east of the Missouri River, which aligned with portions of the Midwest distributional gap (Figure 1; SDGFP, 2020).

Western South Dakota had a semi‐arid climate, whereas eastern South Dakota had a dry, subhumid climate (NOAA, 2021). Lower precipitation and higher evaporation produced a larger moisture deficit in western South Dakota than in eastern South Dakota. The Black Hills was a notable outlier in western South Dakota, where montane topography and landcover reduced evaporation (Widrlechner, 1999). Located in the Northern Great Plains, South Dakota is broadly characterized as a Great Plains ecosystem (Omernik, 2004). The Black Hills represented an eastern extension of the Rocky Mountains and were markedly different from the rest of South Dakota. Eastern South Dakota has experienced a higher rate of land‐use conversion (from native grasslands to row crop production, e.g., corn, *Zea mays*, and soybean, *Glycine max*) than western South Dakota. Land‐use conversion has been identified as an area of concern regarding the maintenance of native grassland ecosystems (Perkins et al., 2019; Wright & Wimberly, 2013).

### 2.2 Sample collection

South Dakota Game, Fish and Parks officials collected tissue samples from the jaws of harvested bobcats presented for CITES export tags from 2014 to 2019. The samples were individually stored in a silica desiccant at room temperature prior to DNA extraction. The county of harvest was documented for each sample. For counties in southwestern South Dakota, whether the bobcat was harvested in the Black Hills was also documented. Information regarding harvest in the Black Hills allowed us to distinguish “subcounties,” which were the portions of southwestern counties that included, or did not include, the Black Hills. One sample was also collected by SDGFP from an incidentally trapped bobcat from a county in eastern South Dakota without a harvest season.

### 2.3 Laboratory

We processed samples at the Laboratory for Ecological, Evolutionary and Conservation Genetics at the University of Idaho. We extracted samples using the Qiagen DNeasy Blood & Tissue Kit (Qiagen, Valencia, CA, USA), and each extraction included a negative control to monitor for contamination. We initially extracted and amplified samples to perform molecular sex identification (Pilgrim et al., 2005). The DNA extract was archived and stored frozen at −20°C. We subsequently used the archived DNA to genotype samples using primers for nuclear DNA microsatellite loci to investigate the genetic diversity and population genetic structure of bobcats. We screened 26 microsatellite loci developed for domestic cat (*Felis catus*; Appendix S1: Table S1) on a subset of 15 bobcat samples that were collected from counties in western South Dakota spanning from the Missouri River to the Black Hills. We excluded nine loci that failed to amplify, amplified weakly, showed evidence of null alleles, or had other irregularities (e.g., nonspecific peaks) from further consideration; we organized the remaining 17 loci into two multiplexes (Appendix S1: Table S1).

We ran polymerase chain reactions (PCR) using a multi‐tubes approach (Taberlet et al., 1996). Each PCR was set up for a 7 μL total volume using the recommended protocols for Qiagen Multiplex PCR Kit, including Q‐solution (0.70 μL), Master Mix (3.50 μL), forward and reverse primers (Appendix S1: Table S1), 1 μL of nuclear DNA extract, and RNase‐free water to make up the final volume. We performed PCR on a Bio‐Rad C1000 Touch 96 Well PCR Thermal Cycler (Bio‐Rad, Hercules, CA, USA) with optimized thermal profiles for each multiplex (Appendix S1: Table S2). We combined LIZ 500 size standard (0.15 μL; Applied Biosystems, Inc., Foster City, CA, USA) and 10 μL formamide with 1 μL of the PCR product. We visualized PCR products with an ABI 3130xl (Applied Biosystems, Inc., Foster City, CA, USA) and scored alleles with GeneMapper Software 6 (Thermo Fisher Scientific, Waltham, MA, USA).

We initially performed PCR amplification of the DNA of each sample in duplicate to minimize the influence of genotyping errors. For each sample, we confirmed alleles at a particular locus for homozygous or heterozygous genotypes if they were observed across ≥2 replicates (i.e., a consensus genotype; Broquet & Petit, 2004). Although it is more common to require that an allele be observed ≥2 times for heterozygous genotypes and ≥3 times for homozygous genotypes (Lonsinger & Waits, 2015), this is usually necessary because of the poorer quality and limited amount of DNA collected through noninvasive genetic sampling (e.g., feces; Broquet & Petit, 2004). Given our use of tissue samples, observing an allele twice was sufficient to establish a consensus genotype. If the scoring of the initial replicates failed to produce a consensus genotype, we then amplified and scored additional replicates as necessary. We used ConGenR in program R to compare replicates, establish consensus genotypes, and calculate genotyping error rates (Lonsinger & Waits, 2015; R Core Team, 2020).

### 2.4 Population genetic structure

Both sample size and the number of loci analyzed can have a strong influence on the power to correctly identify genetic patterns (Landguth et al., 2012). To determine the minimum number of loci over which complete consensus genotypes were required for a sample to be included in the genetic structure analyses, we used GenAlEx v6.503 to calculate the probability of identity among siblings (*p*_(ID)sibs_) (Peakall & Smouse, 2006, 2012; Waits et al., 2001). Individuals analyzed in population clustering analyses should be unrelated, and there is strong evidence that the inclusion of closely related individuals may lead to biased inferences and support for structure when none exists (Anderson & Dunham, 2008; Falush et al., 2003; Hubisz et al., 2009; Rodríguez‐Ramilo & Wang, 2012). We used GenAlEx to estimate relatedness using the Queller and Goodnight (1989) method, which is robust with multilocus testing and does not require random mating in the population (Van De Casteele et al., 2001). We parsimoniously removed individuals that shared pairwise relatedness values ≥0.45 (first‐order relatives i.e., parent‐offspring or full siblings; Viricel & Rosel, 2014).

We evaluated population genetic structure using two Bayesian clustering methods implemented in the programs Structure v2.3.4 (hereafter, structure; Pritchard et al., 2000) and Bayesian Analysis of Population Structure v6.0 (hereafter, BAPS; Cheng et al., 2011; Corander et al., 2006; Corander, Marttinen, et al., 2008). The range of genetically distinct clusters (*K*) considered in Bayesian clustering analyses must be large enough to capture the true value of *K*. Considering the diversity of ecosystems used by bobcats in South Dakota and landscape features (e.g., linear barriers) that may impede dispersal, we considered *K* = 1–20 for structure analyses. structure analyses included 250,000 burn‐ins and 500,000 Markov Chain Monte Carlo repetitions per run using the admixture and correlated allele models (Falush et al., 2003). We initially ran an aspatial analysis in structure for 15 independent batches at each value of *K*. We then repeated the structure analysis in a spatially implicit formulation using dummy variables to code samples into a priori spatial groups (*n* = 36) by sampling a county or subcounty (i.e., the highest spatial resolution of data available for each sample). Spatially implicit structure models incorporating sampling area information are better suited to detect weak patterns of genetic structure, but ignore sampling information when the structure is unrelated to the sampling area (Hubisz et al., 2009). To interpret the structure results, we considered the highest mean log likelihood, *L*(*K*), for each value of *K* in the range for *K* (Pritchard et al., 2000). We also considered the second‐order rate of change in *L*(*K*) of each successive value of *K* (i.e., Δ*K*), which is commonly used to interpret structure results and, in most cases, correctly infers the value of *K* (Evanno et al., 2005). We used Structure Harvester (Earl & von Holdt, 2012) to plot the mean *L*(*K*) estimates and Δ*K* at each *K*. structure also returns individual ancestry values (*q* values), which estimate the proportion of ancestry that is attributable to each inferred genetic cluster. The *q* values for each *K* were selected from the run that had the highest estimated log likelihood of the data among the 15 independent runs in each batch. Individuals were assigned to the genetic clusters for which they had the highest *q* values.

We also investigated the genetic structure of bobcats using BAPS. Aligning with the range of *K* considered in our structure analyses, we set the maximum *K =* 20. We first ran BAPS with the “Clustering of Individuals” model, which was similar to the spatially implicit analysis in structure and included the same samples and sampling information (i.e., spatial groupings). We also ran BAPS using the ‘Spatial Clustering of Individuals’ model, which can consider spatial coordinates in a spatially explicit analysis (Cheng et al., 2013; Corander, Sirén, & Arjas, 2008). Although unique spatial coordinates were not available for each sample, we generated unique spatial coordinates for each sample to run the “Spatial Clustering of Individuals” model in BAPS (sensu Reding et al., 2013). Specifically, we identified centroids for each spatial group using the mean center processing tool in ArcPro v2.4.0 (Esri, Redlands, CA, USA) and generated a random point for each sample within 250 m of the sample's group centroid. We used these spatial coordinates for each sample to perform the spatially explicit analysis with BAPS. For each BAPS analysis, we used 80 independent batches. BAPS stored results from each run and returned the most likely value of *K* as indicated by the *L*(*K*).

To better discern patterns of agreement or conflict among the two structure and two BAPS analyses, we mapped the results of each analysis by classifying each spatial group by the genetic cluster to which the majority of the individuals sampled in that group were assigned. Additionally, we visualized the proportion of individuals from each spatial group that were assigned to each genetic cluster with pie charts. GenAlEx was used to calculate differentiation statistics (*F*_ST_ and $G_{\mathrm{ST}}^{\prime \prime }$), as well as associated *p*‐values via permutation, between pairs of clusters inferred by each Bayesian clustering analysis (Meirmans & Hedrick, 2011; Wright, 1965). We quantified population differentiation between populations with both *F*_ST_ and $G_{\mathrm{ST}}^{\prime \prime }$. We used *F*_ST_ due to historical precedence in the literature, whereas $G_{\mathrm{ST}}^{\prime \prime }$ offers improvements in inference when working with highly variable markers (e.g., microsatellites) and a small number of sampled populations (Meirmans & Hedrick, 2011; Whitlock, 2011).

### 2.5 Genetic diversity

We calculated genetic diversity metrics at each of the 17 loci across all individuals sampled and within each cluster inferred by our Bayesian clustering analyses, unless otherwise noted. To account for multiple comparisons, we used sequential Bonferroni corrections in all evaluations of significance (Rice, 1989). We used the genepop package in Program R (R Core Team, 2020; Raymond & Rousset, 1995) to calculate pairwise linkage disequilibrium, Hardy–Weinberg equilibrium (HWE), and Weir and Cockerham's (1984) inbreeding coefficient (*F*_IS_; a measure of deviation from random mating that determined the extent to which deviations were positive or negative). Evidence of linkage disequilibrium or deviations from HWE at loci in the total sample may reflect a Wahlund effect and indicate cryptic genetic structure (Allendorf et al., 2013). We used Fstat v2.9.4 (Goudet, 1995) to calculate the number of alleles (*A*_N_) and allelic richness (*A*_R_), where allelic richness was a measure of the number of alleles at each locus corrected for sample size. Finally, we used GenAlEx to calculate the observed heterozygosity (*H*_O_) and unbiased expected heterozygosity (*H*_E_), which were the proportion of heterozygotes observed in the sample and the proportion of heterozygotes expected in a panmictic population, respectively.

## 3 RESULTS

### 3.1 Genetic sampling and standard genetic measures

We obtained tissue samples from 1225 bobcats from 2014 to 2019. A combination of 10 loci were required to achieve a *p*_(ID)sib_ < .01 for bobcats, excluding sex identification markers. We removed 10 samples that failed to amplify and 22 samples for which we could not establish complete consensus genotypes at ≥10 loci. For the remaining 1193 samples, we conducted an average of 5.5 replicates/sample (SD = 1.31) to establish consensus genotypes. Genotyping error rates per multilocus genotype were relatively low (overall allelic dropout rate = 0.211%; overall false allele rate = 0.096%). We found 708 pairwise comparisons (0.0016%) between individuals had relatedness values >0.45 and parsimoniously removed the 315 individuals that occurred in the most related pairs until all related pairs had at least one individual removed. For analyses that did not require spatial data, we retained 878 samples (53.4% male, 45.3% female, and 1.3% unknown) from 31 counties (23 samples did not have county information; Figure 1). Finally, when applying Bayesian clustering analyses where the sampling area was considered, we removed 23 samples for which the county of harvest was unknown.

For the total sample of 878 bobcats, the mean number of alleles (*A*_N_) across loci was 10.9 (SD = 3.6, range = 7–19; Table 1). The values of *F*_IS_ were positive and *H*_O_ < *H*_E_ for all loci, with significant departures from HWE in 10 of 17 loci (Table 1). Results were similar when considering only the 855 samples retained for analyses requiring locational data (Appendix S2: Table S1). We found evidence of linkage disequilibrium in 5.9% of the 136 pairwise comparisons.

**TABLE 1:** The number of alleles (*A*_N_), observed heterozygosity (*H*_O_), unbiased expected heterozygosity (*H*_E_), Weir and Cockerham's inbreeding coefficient (*F*_IS_), standard error (SE) for *F*_IS_, and *p*‐value (*p*) for the test of Hardy–Weinberg equilibrium for 17 microsatellite loci amplified and considered in population genetic structure analyses for 878 bobcats sampled in South Dakota (USA) from December 2014 to February 2019; *p*‐values in bold indicate significant departures from Hardy–Weinberg equilibrium after sequential Bonferroni corrections.

Locus	*A*_N_	*H*_O_	*H*_E_	*F*_IS_	SE	*p*
F124	14	0.84	0.86	0.020	.023	.110
F53	16	0.81	0.82	0.011	.002	**.003**
FCA008	8	0.73	0.78	0.055	.000	**.000**
FCA026	15	0.82	0.84	0.022	.001	**.001**
FCA043	7	0.72	0.76	0.046	.011	.091
FCA057	13	0.81	0.85	0.053	.000	**.000**
FCA082	11	0.80	0.83	0.040	.017	.066
FCA090	7	0.70	0.79	0.117	.000	**.000**
FCA096	19	0.80	0.90	0.104	.000	**.000**
FCA098	11	0.52	0.77	0.324	.000	**.000**
FCA117	8	0.75	0.78	0.039	.019	.089
FCA132	8	0.77	0.81	0.047	.023	.407
FCA205	9	0.46	0.74	0.381	.000	**.000**
FCA229	12	0.80	0.81	0.020	.000	**.000**
FCA275	8	0.68	0.70	0.025	.005	.011
FCA391	7	0.71	0.72	0.019	.014	.068
FCA741	12	0.78	0.80	0.020	.000	**.000**
Mean	10.9	0.74	0.80	0.079	.026	—

### 3.2 Population genetic structure analyses

**FIGURE 2:**
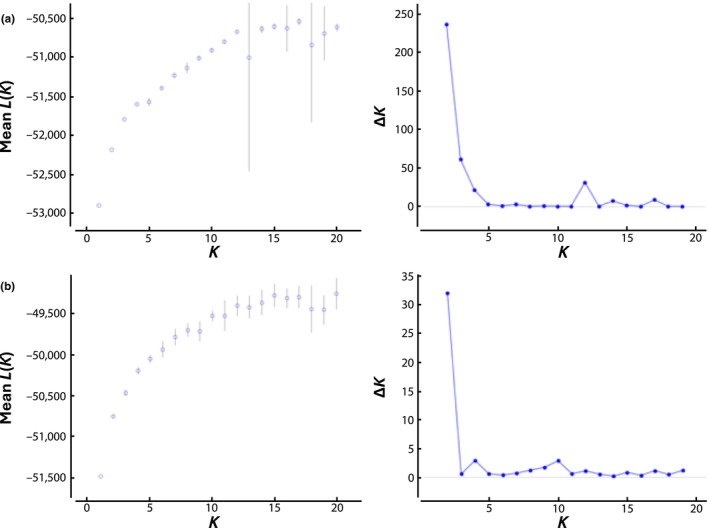
The most likely number of genetic clusters (*K*) of bobcats (*Lynx rufus*) harvested in South Dakota, USA, from December 2014 to February 2019 based on the mean log‐likelihood (*L*[*K*]) ± 1 standard deviation and Δ*K* (the rate of change in *L*[*K*] for each subsequent *K*) using the (a) aspatial and (b) spatially implicit models in the program structure.

All four analyses found support for population genetic structure. The aspatial structure analysis provided support for *K* = 2 clusters based on peaks in Δ*K*, whereas the most supported number of clusters based on *L*(*K*) was more ambiguous (Figure 2a). For the spatially implicit structure analysis, Δ*K* provided support for *K* = 2 and *K =* 4 clusters, but the results based on *L*(*K*) were again more ambiguous (Figure 2b). The results from the BAPS analyses agreed with those of structure. The spatially explicit BAPS analysis indicated *K* = 2 to be the most likely number of clusters, whereas the spatially implicit BAPS analysis indicated *K* = 10. Both Structure analyses also provided support for *K* ≥ 10 clusters. The lack of fine‐scale spatial data for samples limited our ability to confidently interpret results when analyses supported *K* ≥ 10 clusters.

**FIGURE 3:**
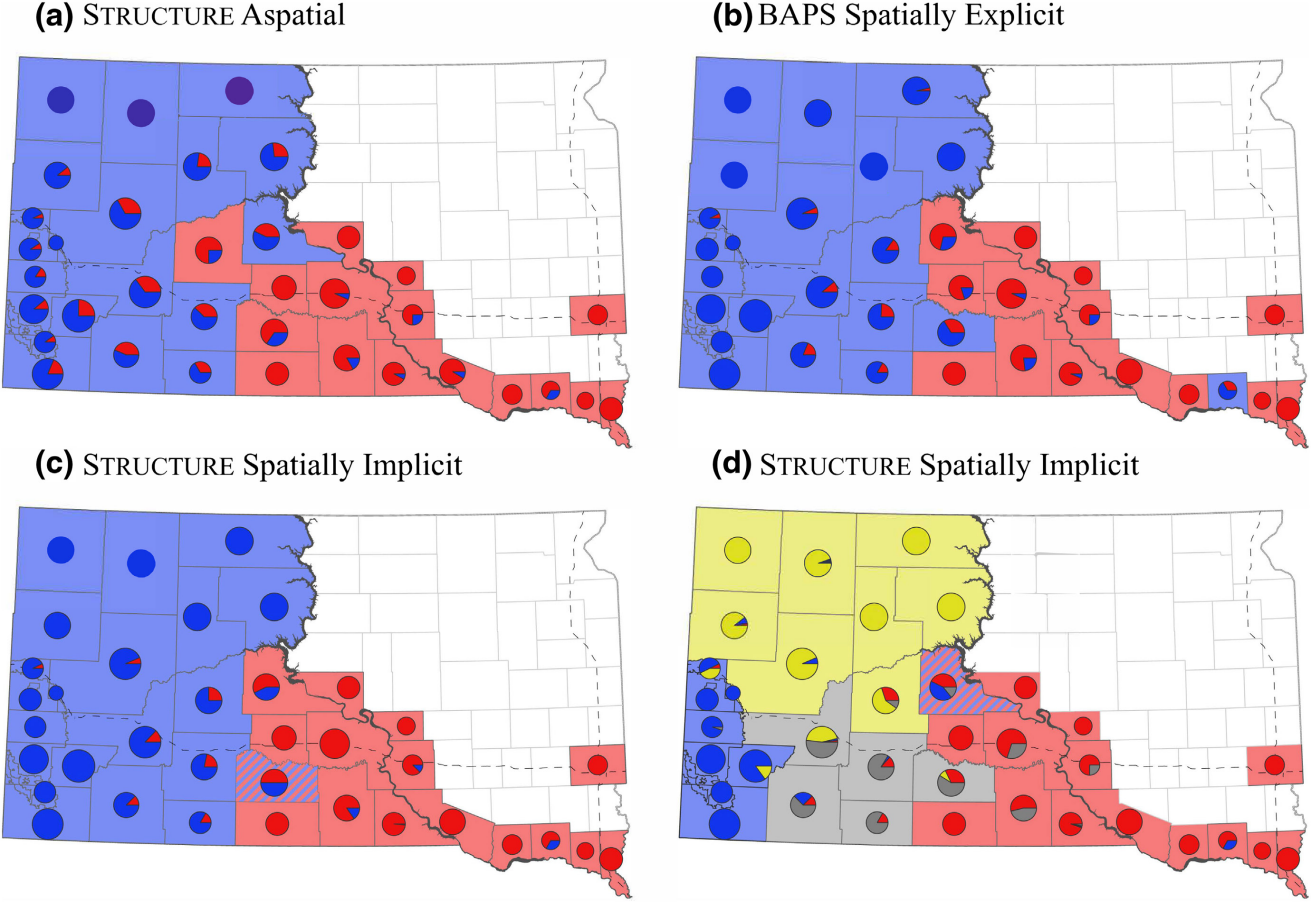
County‐level visualizations of the Bayesian clustering algorithm results supporting two or four genetic clusters for harvested bobcats (*Lynx rufus*) sampled in South Dakota, USA, from December 2014 to February 2019, where each spatial group (county or subcounty) is assigned to the genetic cluster from which the highest proportion of individuals harvested in the group were assigned (as indicated by the pie chart). Results from (a) structure using the aspatial model indicated two genetic clusters (blue = western cluster; red = eastern cluster), which was supported by (b) the spatially explicit model in Bayesian Analysis of Population Structure (BAPS) and (c) the spatially implicit model in structure. The (d) spatially implicit model in structure also supported four genetic clusters, including northwestern (yellow), Black Hills (blue), southcentral (gray), and eastern (red) clusters. Aspatial analysis included 878 bobcats, whereas spatial analyses were restricted to 855 bobcats with sufficient locational data. Dashed lines indicate major (interstate) highways, whereas the variable‐width gray line bisecting South Dakota from north to south indicates the Missouri River.

Mapping the results for each analysis supporting *K* = 2 clusters indicated high levels of geographic congruence among analyses and indicated a western cluster, an eastern cluster, and a contact zone (i.e., an area where two genetic clusters meet) in southcentral South Dakota west of the Missouri River (Figure 3). Mapping the results of the spatially implicit structure analysis for *K* = 4 clusters indicated that the eastern most cluster aligned geographically with the counties within the eastern cluster identified at *K* = 2 of the same analysis. The remaining three clusters were nested within the western cluster identified by the spatially implicit structure results for *K* = 2 and aligned with the northwestern, southcentral, and Black Hills regions of South Dakota (Figure 3).

In the three analyses supporting *K* = 2 clusters, measures of *F*_ST_ and $G_{\mathrm{ST}}^{\prime \prime }$ suggested the eastern and western clusters were significantly differentiated from one another (Table 2). Estimated $G_{\mathrm{ST}}^{\prime \prime }$ values indicated moderate levels of differentiation (0.05–0.15; Wright, 1978), with estimated differentiation being lowest for the aspatial structure analysis compared to the spatially implicit structure and spatially explicit BAPS analyses (Table 2). For the spatially implicit structure analysis supporting *K* = 4 clusters, *F*_ST_ and $G_{\mathrm{ST}}^{\prime \prime }$ indicated significant differentiation between all four inferred clusters (Table 3). There were moderate levels of differentiation among western clusters (i.e., northwestern, southcentral, and Black Hills) and moderate to strong differentiation between the eastern cluster and each of the three western clusters (Table 3).

**TABLE 2:** Pairwise *F*_ST_ (bottom row) and $G_{\mathrm{ST}}^{\prime \prime }$ (top row) from GenAlEx between *K* = 2 genetic clusters of bobcats sampled in South Dakota (USA) from December 2014 to February 2019 from the aspatial structure, spatially implicit structure, and spatially explicit BAPS analyses; all values were significant at *p* < .001 and differentiation of 0.05–0.15 was considered moderate differentiation (Wright, 1978).

*K*	Aspatial Structure		Spatially implicit Structure		Spatially explicit BAPS	
	Eastern	Western	Eastern	Western	Eastern	Western
Eastern	—	0.105	—	0.115	—	0.119
Western	0.012	—	0.013	—	0.014	—

**TABLE 3:** Pairwise *F*_ST_ (below the diagonal) and $G_{\mathrm{ST}}^{\prime \prime }$ (above the diagonal) from GenAlEx between *K* = 4 genetic clusters of bobcats sampled in South Dakota (USA) from December 2014 to February 2019 from the spatially implicit structure analysis; all values were significant at *p* < .001 and differentiation of 0.05–0.15 was considered moderate differentiation (Wright, 1978).

*K*	Eastern	Northwestern	Southcentral	Black Hills
Eastern	—	0.149	0.137	0.153
Northwestern	0.018	—	0.074	0.073
Southcentral	0.017	0.010	—	0.083
Black Hills	0.018	0.009	0.010	—

For analyses resulting in the partition of bobcats into *K* = 2 and *K =* 4 clusters, mean *H*_O_ within clusters ranged from 0.721 to 0.745 and 0.722 to 0.752, respectively, and mean *H*_O_ was consistently less than *H*_E_ (Table 4). Compared to the total sample, departures from HWE and proportion of loci comparisons with linkage disequilibrium were reduced within inferred clusters across analyses. When analyses supported *K* = 2 clusters, departures in HWE were reduced to only 4–7 loci (i.e., 23.5%–41.2% of loci) within inferred clusters, and the proportion of pairwise comparisons among loci with evidence of linkage disequilibrium was reduced to 0.0%–2.9% (Appendix S2: Table S2). Within‐cluster departures in HWE were reduced further to 2–4 loci (i.e., 11.8%–23.5% of loci) when considering analyses supporting *K* = 4 clusters (Appendix S2: Table S2), though two loci departed from HWE across all clusters (Appendix S2: Table S3).

**TABLE 4:** Per cluster (*K*) mean allelic richness (*A*_R_), observed heterozygosity (*H*_O_), unbiased expected heterozygosity (*H*_E_), Weir and Cockerham's inbreeding coefficient (*F*_IS_), as well as the standard error (SE) of *F*_IS_ for bobcat samples collected in South Dakota (USA) from December 2014 to February 2019 from aspatial structure analysis results for *K* = 2, spatially implicit structure analysis results for *K* = 2 and *K* = 4, and spatially explicit BAPS results for *K* = 2.

Analysis	*K*	Cluster	*A*_R_	*H*_O_	*H*_E_	*F*_IS_	SE
Aspatial structure	2	East	10.1	0.722	0.776	0.074	.023
		West	10.0	0.745	0.797	0.065	.028
Spatially implicit structure	2	East	9.8	0.724	0.775	0.069	.023
		West	9.7	0.739	0.794	0.071	.027
Spatially implicit structure	4	East	9.2	0.722	0.769	0.063	.022
		Northwest	8.8	0.726	0.778	0.067	.029
		Southcentral	8.9	0.743	0.789	0.061	.025
		Black Hills	9.2	0.752	0.798	0.059	.029
Spatially explicit BAPS	2	East	9.3	0.721	0.773	0.069	.024
		West	9.7	0.739	0.795	0.071	.027

## 4 DISCUSSION

Our findings indicated that despite having a broad distribution, high movement capacity, and generalist tendencies, bobcats in South Dakota were not a single panmictic population. Rather, bobcats were genetically differentiated into eastern and western populations, with further preliminary evidence of cryptic population genetic structure within the western population. We found no evidence that large rivers or highways influenced gene flow, but population genetic structure appeared to align with broad‐scale habitat‐specific breaks. We demonstrated that, for an elusive species, samples from harvested animals provided the broad‐scale sampling required to efficiently and effectively identify genetic structure among populations.

Bobcats recolonized central North America from historical Pleistocene refugia in eastern and western portions of the continent, and the population genetic structure of bobcats has been maintained longitudinally along the Great Plains, which has been identified as a suture zone (or secondary contact zone; Reding et al., 2012). In South Dakota, the location of the suture zone aligned closely with the boundary between our eastern and western clusters. Environmental gradients can also produce genetic structure (Wang & Bradburd, 2014), and Reding et al. (2012) found that variation in precipitation explained a significant portion of bobcat population genetic structure. Thus, the differentiation between eastern and western clusters that we observed along the Great Plains suture zone could also be influenced by acute differences in precipitation between eastern and western South Dakota.

Although linear features have the potential to limit gene flow, their influence may be context dependent. For example, telemetered bobcat movements were inhibited by large rivers in Illinois (Nielsen & Woolf, 2003), but not in Indiana (Johnson et al., 2010). Reding et al. (2012) did not find evidence that the Mississippi River was a significant barrier to gene flow for bobcats, but they excluded samples from around the northern headwater portions of the river in Minnesota and Wisconsin. We did not find support for our hypothesis that the Missouri River acted as a barrier to gene flow in bobcats. Rather, the eastern cluster that we observed was found on both sides of the Missouri River. Bobcats in agricultural landscapes tend to select for riparian areas, which may provide high‐quality cover and prey, while also serving as movement corridors (Hilty & Merenlender, 2004; Kolowski & Woolf, 2002). Furthermore, in South Dakota, the Missouri River freezes in winter, which coincides with juvenile dispersal and may facilitate dispersal or extraterritorial movements when prey is scarce (Knick, 1990). Highways may also act as a barrier to gene flow for bobcats in some situations (Riley et al., 2006), but not others (Millions & Swanson, 2007). Bobcat gene flow was significantly restricted by a large (10–12 lanes) heavily trafficked (>150,000 vehicles/day) highway in California (Riley et al., 2006). We did not observe patterns of population genetic structure that aligned with an east–west 4‐lane highway (i.e., Interstate 90) that crossed our sampling extent. The relatively low traffic volume (~21,000 vehicles/day; Highway Performance Monitoring System, 2011) may not have restricted bobcat gene flow.

In the absence of physical barriers to movement, fine‐scale population genetic structure for generalist carnivores has been attributed to environmental differences and intraspecific variability in habitat proclivities or specializations (Bolnick et al., 2007), leading to isolation by environment (Wang & Bradburd, 2014). The population structure of gray wolves (*Canis lupus*) was correlated with climate, habitat types, and prey specialization (Pilot et al., 2006). Similarly, coyotes (*Canis latrans*) exhibited fine‐scale genetic structure boundaries consistent with habitat‐specific breaks (Sacks et al., 2004). Bobcat populations have been panmictic in some areas (e.g., Croteau et al., 2010) or structured by physical barriers (e.g., mountains, Reding et al., 2013; highways, Riley et al., 2006; and anthropogenic landscapes, Smith et al., 2020). More recently, Cancellare et al. (2021) provided evidence that bobcat populations can be structured via isolation by environment at multiple scales. Genetic clusters associated with our results for *K* = 4 were geographically contiguous, congruent with broader patterns seen at *K* = 2, and captured a pattern of bobcat population genetic structure that aligned with structural landscape changes (i.e., level III ecoregions); the eastern cluster was primarily in the Northern and Northwestern Glaciated Plains; the northwestern cluster was primarily in the Northwestern Great Plains; the southcentral cluster was characterized by the High Plains and Nebraska Sand Hills; and the Black Hills cluster was centered on the Middle Rockies (U.S. Environmental Protection Agency, 2013). Consequently, isolation by environment may be driving fine‐scale genetic structure in the bobcat populations in western South Dakota. Patterns of genetic diversity and decreases in the observed departures from Hardy–Weinberg and linkage equilibrium with *K* = 4 were consistent with a Wahlund effect (Allendorf et al., 2013). Still, results associated with *K* = 4 should be viewed as preliminary, as these patterns were detected by only a single analysis.

Genetic sampling of wildlife can present challenges. For bobcats, hair snares have low effectiveness, and efficient fecal DNA sampling requires the use of scat detection dogs (Long et al., 2009), limiting cost efficiency for broad‐scale population assessments. Sampling harvested individuals can be an efficient alternative sampling source, but these samples are potentially susceptible to incomplete participation and reporting (Schmidt et al., 2015), as well as sex‐ and age‐biased harvest (Allen et al., 2018). Our study benefited from state regulations that required harvested bobcats to be checked by wildlife management officials, which minimized nonreporting. For bobcats, sex and age bias in harvest tends to be greater with hunting (e.g., with hounds) than trapping (Allen et al., 2018), and >75% of bobcat samples collected during our study were harvested by trappers (SDGFP, 2021); additionally, our sample had a fairly even sex ratio.

In the United States, landscapes are dominated by private lands (i.e., 74% of states are ≥70% private lands; Morgan et al., 2019), and broad‐scale monitoring of wildlife populations may be limited by access to private lands in many areas; therefore, most wildlife management efforts have occurred on public lands (Daley et al., 2004). In areas like South Dakota that are predominantly private lands (Morgan et al., 2019), using harvested samples to monitor populations may facilitate broader spatial sampling than alternative monitoring approaches that require obtaining permissions to access private lands. Although, the majority of hunters and trappers harvest game on private lands (85%; Morgan et al., 2019), the spatial distribution of harvest can be nonrandom, and locational data for harvested samples may be reported on a relatively course scale.

There can be substantial spatial variation in harvest rates (and therefore samples) due to differences in harvest regulations (e.g., bag limits) across a study area. The distribution of geographic sampling may confound our ability to disentangle the influences of population genetic structure and isolation by distance. Failure to account for underlying patterns of isolation by distance can lead to spurious identification of genetic clusters where they do not actually occur when employing Bayesian clustering algorithms, such as those implemented in structure (Meirmans, 2012; Schwartz & McKelvey, 2009). When underlying patterns of isolation by distance exist, some have argued that uneven sampling can lead to spurious genetic clusters (Turbek et al., 2023), whereas others have suggested that even sampling may introduce more bias in the estimation of genetic clusters (Frantz et al., 2009). We lacked fine‐scale locational data for harvest samples and were therefore unable to fully characterize the evenness of our sampling or explicitly investigate patterns of isolation by distance. Practitioners intending to use harvest samples to evaluate genetic structure and landscape genetic hypotheses could benefit from ensuring precise spatial data are collected for each sample, enabling a formal assessment of isolation by distance and its potential influence on observed patterns of population structure. Despite these limitations, we followed the recommendations to use clustering approaches that account for the spatial location of samples (Meirmans, 2012) by employing the spatially explicit model in BAPS that has been demonstrated to be robust to datasets with underlying patterns of isolation by distance (Frantz et al., 2006).

The relative intensity of sampling among genetic clusters may also influence the detection of population genetic structure. Kalinowski (2011) and Puechmaille (2016) suggested that unbalanced sample sizes may result in estimation errors for the number of populations with aspatial Bayesian clustering algorithms. Wang (2017) concluded that these concerns were inflated, though, and that estimation error with unbalanced sample sizes was a consequence of the model (i.e., the aspatial model) and the relative magnitude of imbalance between sampled populations. Wang (2017) demonstrated that the aspatial ancestry model produced reliable inferences when the sampling of populations was balanced (i.e., sample size ratios ≤5:1) but not when the sampling was unbalanced (i.e., sample size ratios >5:1). In contrast, spatial models produced reliable inferences even with highly unbalanced sample sizes (e.g., sample size ratios ≤38:1). Although our sample sizes appear disproportionate across counties (or subcounties), our population‐level sample sizes were within the ratio values identified as balanced (Wang, 2017), and the number of individuals per inferred cluster well exceeded the number needed to adequately characterize allele frequencies for population genetic studies (i.e., 25–30; Hale et al., 2012). When population delineation is a primary objective, spatially balancing sampling may not always result in relatively balanced sampling among inferred genetic clusters. Consequently, the application of spatial models can help eliminate bias introduced by unbalanced sampling of populations and we have greater confidence in the patterns resulting from our spatial models than those from the aspatial model.

Harvest units that align with population boundaries can help minimize the risks of overharvest, without introducing unnecessary monitoring or management costs (Allendorf et al., 2013; Rosenberry & Diefenbach, 2019; Taylor & Dizon, 1999). Our results for *K* = 2 indicated that the Missouri River did not align with population boundaries in the southern half of South Dakota; including counties immediately west of the Missouri River and south of the Oahe Dam (an impoundment of the Missouri River in central South Dakota) as part of the eastern harvest unit would better align units with the boundaries of genetic clusters. Thus, two bobcat harvest units in South Dakota could offer a feasible approach that provides managers with sufficient population‐level control to regulate harvest while maintaining genetic diversity (Allendorf et al., 2013).

## AUTHOR CONTRIBUTIONS

**Stuart C. Fetherston:** Data curation (lead); formal analysis (lead); investigation (lead); methodology (equal); visualization (lead); writing – original draft (lead); writing – review and editing (equal). **Robert C. Lonsinger:** Conceptualization (lead); data curation (supporting); formal analysis (supporting); funding acquisition (equal); methodology (equal); project administration (lead); resources (equal); supervision (lead); writing – original draft (supporting); writing – review and editing (lead). **Lora B. Perkins:** Funding acquisition (equal); project administration (supporting); supervision (supporting); writing – review and editing (equal). **Chadwick P. Lehman:** Conceptualization (supporting); funding acquisition (equal); resources (equal); writing – review and editing (equal). **Jennifer R. Adams:** Data curation (supporting); formal analysis (supporting); investigation (supporting); methodology (equal); writing – review and editing (equal). **Lisette P. Waits:** Conceptualization (supporting); formal analysis (supporting); funding acquisition (equal); methodology (equal); project administration (supporting); resources (equal); writing – review and editing (equal).

## CONFLICT OF INTEREST STATEMENT

The authors declare no conflict of interest or competing interests.

## Supporting information


Appendix S1

## Data Availability

Data are available from the Open PRAIRIE data repository at https://openprairie.sdstate.edu/nrm_datasets/11.
