# Supplementary material for: Genetic analysis of harvest samples reveals population structure in a highly mobile generalist carnivore
Source: Ecol Evol. 2024 May 23;14(5):e11411. doi: 10.1002/ece3.11411 (PMC11116766; doi:10.1002/ece3.11411)
Supplement: Supplementary file 1 — Appendix S1 [file ECE3-14-e11411-s001.docx]

# Appendix S1. Microsatellite multiplex and polymerase chain reaction (PCR) conditions

**Table S1.** Microsatellite loci screened for use in multiplexes for bobcat samples collected in South Dakota (USA) from December 2014–February 2019 and the final concentration (μM) for the 17 nuclear DNA microsatellite loci selected for two multiplexes (Multiplex 1 and Multiplex 2). All loci were tested with forward and reverse primers, initially at a volume of 0.09 μL and adjusted as needed in each multiplex. Loci with multiple fluorescent dyes listed were tested for each dye and the dye that was accepted is indicated in bold. All primers were used at a concentration of 10 μM. Removed indicates loci that were excluded from further consideration.

| **Multiplex** | **Locus** | **Dye** | **Final Concentration (μM)** |
| --- | --- | --- | --- |
| Multiplex 1 | F124^1,2^ | PET | 0.04 |
|  | FCA026^1^ | PET | 0.06 |
|  | FCA043^1^ | NED | 0.04 |
|  | FCA057^1^ | VIC | 0.07 |
|  | FCA082^1^ | PET | 0.14 |
|  | FCA090^1^ | 6-FAM | 0.10 |
|  | FCA096^1^ | 6-FAM | 0.11 |
|  | FCA098^1^ | **VIC** / 6-FAM | 0.04 |
|  | FCA132^1^ | NED | 0.11 |
|  | FCA229^1^ | 6-FAM | 0.06 |
| Multiplex 2 | F53^1,2^ | NED | 0.29 |
|  | FCA008^1^ | 6-FAM | 0.09 |
|  | FCA117^1^ | 6-FAM | 0.36 |
|  | FCA205^1^ | VIC / **6-FAM** | 0.29 |
|  | FCA275^1^ | VIC | 0.13 |
|  | FCA391^1,2^ | NED | 0.17 |
|  | FCA741^2^ | PET | 0.06 |
| Removed | F41^1,2^ | NED | - |
|  | F85^1,2^ | VIC | - |
|  | F98^1,2^ | 6-FAM | - |
|  | FCA045^1^ | PET | - |
|  | FCA075^1^ | VIC | - |
|  | FCA124^1^ | NED | - |
|  | FCA126^1^ | PET | - |
|  | FCA166^3^ | VIC | - |
|  | FCA225^1^ | PET | - |

Reference: ^1^Menotti-Raymond et al. 1999; ^2^Menotti-Raymond et al. 2005; ^3^Culver et al. 2001.

**Table S2.** Polymerase chain reaction thermal profiles for each of two multiplexes used for bobcat samples collected in South Dakota (USA) from December 2014–February 2019.

| **Multiplex** | **Step** | **Temperature (**°C) | **Duration** |
| --- | --- | --- | --- |
| Multiplex 1 | Initial denaturation | 95° C | 0:15:00 |
|  | Touchdown | – | 13 Cycles |
|  | Denaturation | 94° C | 0:00:30 |
|  | Annealing | 60° C – 0.8° C | 0:01:30 |
|  | Extension | 72° C | 0:01:00 |
|  | Cycling | – | 22 Cycles |
|  | Denature | 94° C | 0:00:30 |
|  | Annealing | 50° C | 0:01:30 |
|  | Extension | 72° C | 0:01:00 |
|  | Final extension | 60° C | 0:30:00 |
|  | Cooldown | 04° C | 0:10:00 |
| Multiplex 2 | Initial denaturation | 95° C | 0:15:00 |
|  | Touchdown | – | 20 Cycles |
|  | Denaturation | 94° C | 0:00:30 |
|  | Annealing | 62° C – 0.6° C | 0:01:30 |
|  | Extension | 72° C | 0:01:00 |
|  | Cycling | – | 25 Cycles |
|  | Denature | 94° C | 0:00:30 |
|  | Annealing | 50° C | 0:01:30 |
|  | Extension | 72° C | 0:01:00 |
|  | Final extension | 60° C | 0:30:00 |
|  | Cooldown | 04° C | 0:10:00 |

# LITERATURE CITED

Culver, M., M. A. Menotti-Raymond, and S. J. O’Brien. 2001. Patterns of size homoplasy at 10 microsatellite loci in pumas (*Puma concolor*). Molecular Biology and Evolution 18:1151–1156.

Menotti-Raymond, M., V. A. David, L. A. Lyons, A. A. Schäffer, J. F. Tomlin, M. K. Hutton, and S. J. O’Brien. 1999. A genetic linkage map of microsatellites in the domestic cat (*Felis catus*). Genomics 57:9–23.

Menotti-Raymond, M. A., V. A. David, L. L. Wachter, J. M. Butler, and S. J. O’Brien. 2005. An STR forensic typing system for genetic individualization of domestic cat (*Felis catus*). Journal of Forensic Sciences 50:1061–1070.

# Appendix S2. Genetic diversity and population genetic structure estimates

**Table S1.** The number of alleles (A_N_), observed heterozygosity (H_O_), unbiased expected heterozygosity (H_E_), Weir and Cockerham's inbreeding coefficient (*F_IS_*), standard error (*SE*) for *F_IS_*, and *P*-value (*P*) for the test of Hardy-Weinberg equilibrium for 17 microsatellite loci amplified and considered in population genetic structure analyses for 855 bobcats sampled in South Dakota (USA) from December 2014–February 2019 that had sufficient locational data. *P*-values in bold indicate significant departures from Hardy-Weinberg equilibrium after sequential Bonferroni corrections.

| **Locus** | **A_N_** | **H_O_** | **H_E_** | ***F_IS_*** | ***SE*** | ***P*** |
| --- | --- | --- | --- | --- | --- | --- |
| F124 | 14 | 0.84 | 0.86 | 0.022 | 0.018 | 0.130 |
| F53 | 16 | 0.81 | 0.82 | 0.011 | 0.007 | 0.014 |
| FCA008 | 8 | 0.73 | 0.78 | 0.061 | 0.000 | **0.000** |
| FCA026 | 15 | 0.82 | 0.84 | 0.021 | 0.002 | **0.002** |
| FCA043 | 7 | 0.72 | 0.76 | 0.046 | 0.024 | 0.130 |
| FCA057 | 13 | 0.81 | 0.85 | 0.053 | 0.003 | **0.003** |
| FCA082 | 11 | 0.80 | 0.83 | 0.042 | 0.016 | 0.062 |
| FCA090 | 7 | 0.70 | 0.79 | 0.121 | 0.000 | **0.000** |
| FCA096 | 19 | 0.80 | 0.89 | 0.103 | 0.000 | **0.000** |
| FCA098 | 11 | 0.52 | 0.77 | 0.324 | 0.000 | **0.000** |
| FCA117 | 8 | 0.74 | 0.78 | 0.044 | 0.013 | 0.048 |
| FCA132 | 8 | 0.77 | 0.81 | 0.047 | 0.026 | 0.520 |
| FCA205 | 9 | 0.47 | 0.75 | 0.374 | 0.000 | **0.000** |
| FCA229 | 12 | 0.79 | 0.81 | 0.022 | 0.000 | **0.000** |
| FCA275 | 8 | 0.68 | 0.70 | 0.022 | 0.003 | 0.007 |
| FCA391 | 7 | 0.70 | 0.72 | 0.027 | 0.009 | 0.057 |
| FCA741 | 12 | 0.78 | 0.80 | 0.020 | 0.000 | **0.000** |
| Mean | 10.9 | 0.73 | 0.80 | 0.080 | 0.026 | **-** |

**Table S2.** Per cluster (*K*) proportion of 17 microsatellite loci (%Loci) deviating from Hardy-Weinberg equilibrium and proportion of 136 pairwise comparisons (%Comparisons) among loci with evidence of linkage disequilibrium for bobcat samples collected in South Dakota (USA) from December 2014–February 2019 when pooled into *K* =1 cluster or separated based on aspatial Structure analysis results for *K* = 2, spatially implicit Structure analysis results for *K* = 2 and *K* = 4, or spatially explicit BAPS results for *K* = 2.

| **Analysis** | ***K*** | **Cluster** | **%Loci** | **%Comparisons** |
| --- | --- | --- | --- | --- |
| Pooled samples | 1 | South Dakota | 58.8% | 5.9% |
| Aspatial Structure | 2 | East | 35.3% | 2.2% |
|  |  | West | 35.3% | 2.9% |
| Spatially implicit Structure | 2 | East | 23.5% | 0.7% |
|  |  | West | 41.2% | 2.2% |
| Spatially implicit Structure | 4 | East | 23.5% | 2.9% |
|  |  | Northwest | 23.5% | 1.5% |
|  |  | Southcentral | 11.8% | 0.0% |
|  |  | Black Hills | 17.6% | 0.0% |
| Spatially explicit BAPS | 2 | East | 29.4% | 0.0% |
|  |  | West | 41.2% | 1.5% |

**Table S3.** *P*-values for the tests of Hardy-Weinberg equilibrium for 17 microsatellite loci amplified for 855 bobcat samples collected in South Dakota (USA), 2014–2019, and assigned to the eastern, northwestern, southcentral, and Black Hills clusters by the spatially implicit Structure analysis supporting *K* = 4 genetic clusters. Bold indicates significant departures from Hardy-Weinberg equilibrium following sequential Bonferroni corrections.

| **Loci** | **Eastern** | **Northwestern** | **Southcentral** | **Black Hills** |
| --- | --- | --- | --- | --- |
| F124 | 0.348 | 0.056 | 0.850 | 0.579 |
| F53 | 0.278 | 0.098 | 0.392 | 0.195 |
| FCA008 | 0.074 | 0.546 | 0.010 | 0.203 |
| FCA026 | 0.087 | 0.008 | 0.692 | 0.034 |
| FCA043 | 0.519 | 0.129 | 0.103 | 0.345 |
| FCA057 | 0.198 | 0.011 | 0.183 | 0.157 |
| FCA082 | 0.594 | 0.855 | 0.692 | 0.967 |
| FCA090 | **0.001** | **0.002** | 0.032 | 0.362 |
| FCA096 | 0.104 | **0.000** | 0.201 | **0.000** |
| FCA098 | **0.000** | **0.000** | **0.000** | **0.000** |
| FCA117 | 0.247 | 0.219 | 0.019 | 0.478 |
| FCA132 | 0.787 | 0.698 | 0.249 | 0.815 |
| FCA205 | **0.000** | **0.000** | **0.000** | **0.000** |
| FCA229 | **0.000** | 0.005 | 0.695 | 0.047 |
| FCA275 | 0.244 | 0.063 | 0.273 | 0.632 |
| FCA391 | 0.508 | 0.063 | 0.853 | 0.074 |
| FCA741 | 0.077 | 0.004 | 0.012 | 0.592 |
